# Closer view of antennal sensory organs of two *Leptoglossus* species (Insecta, Hemiptera, Coreidae)

**DOI:** 10.1038/s41598-023-27837-4

**Published:** 2023-01-12

**Authors:** Artur Taszakowski, Adrian Masłowski, Kent M. Daane, Jolanta Brożek

**Affiliations:** 1 Institute of Biology, Biotechnology and Environmental Protection, Faculty of Natural Sciences, University of Silesia in Katowice, Bankowa 9, 40-007 Katowice, Poland; 2 Department of Environmental Science, Policy, and Management, University of California Berkeley, Berkeley, CA 94720-3114 USA

**Keywords:** Structural biology, Zoology

## Abstract

Detailed description of antennal sensory organs of *Leptoglossus occidentalis* Heidemann, 1910 (Insecta: Hemiptera: Heteroptera: Coreidae) and a comparison with *L. zonatus* (Dallas, 1852) are presented. A novel approach that combines the advantages of field emission scanning electron microscopy (FE-SEM) and atomic force microscope (AFM) was used to detail micromorphological structures. A simplified classification system for sensilla that eliminates the subjective aspects of morphology, such as their shape, is proposed. Fourteen sensory organs have been classified into three main groups: (a) aporous sensilla with a flexible socket, (b) porous sensilla with a flexible socket and (c) porous sensilla with an inflexible socket. A large variety of sensory organs (nine types) with olfactory functions are described. The antennal sensory organs have been recognized as one of the factors responsible for the evolutionary success of *Leptoglossus* spp. and their status as important pests and invasive species.

## Introduction

The Heteroptera, called "true bugs", are now recognized as a monophyletic suborder within the order Hemiptera. With more than 45,000 described species, true bugs are part of the most successful radiation of non-endopterygote and nonholometabolous insects. They are a diverse group whose representatives occur in often radically different habitats in all zoogeographical regions^1,2^.

The genus *Leptoglossus* Guérin-Méneville, 1831^3^ belongs to the infraorder Pentatomomorpha Leston, Pedergrast et Southwood, 1954^4^, the superfamily Coreoidea Leach, 1815, the family Coreidae Leach, 1815^5^, the subfamily Coreinae Leach, 1815 and the tribe Anisoscelini Laporte, 1832^6,7^. Coreids, called leaf-footed or squash bugs, are all phytophagous, and live on plants, feeding above ground^2^ in the plant vascular system^8^. The genus *Leptoglossus* includes 62 species^7^ of a neotropical origin, with most species limited to Central and South America^9,10^. A number of *Leptoglossus* species, including both species that are the subject of this paper, are important plant pests^11^.

**Figure 1:**
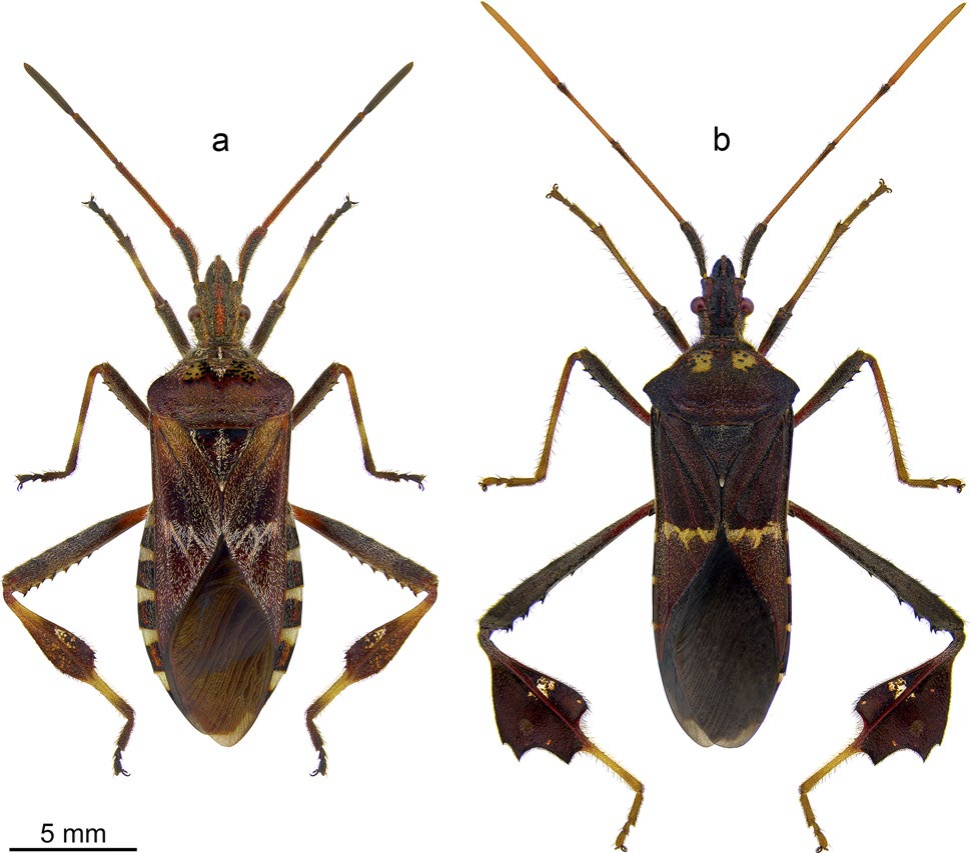
Dorsal habitus of examined species: *L. occidentalis* (**a**) and *L. zonatus* (**b**).

*Leptoglossus occidentalis* Heidemann, 1910^12^ also called the western conifer seed bug (Fig. 1a), a native of western North America, over the last six decades has significantly expanded its range to include eastern North America, Europe, Asia Minor, Eastern Asia, northern Africa, and South America^7,13^. *Leptoglossus occidentalis* exhibits polyphagous habits in both its native and invasive ranges. This species feeds on conifer cones and can be a major pest in conifer seed orchards^14,15^. In Europe, it is of particular concern to growers of edible pine nuts, *Pinus pinea* L.^16^.

*Leptoglossus zonatus* (Dallas, 1852)^17^ or large-legged bug (Fig. 1b), is a polyphagous species found throughout much of the Western Hemisphere^18^. *Leptoglossus zonatus* is an important pest of a wide range of crops in the southern United States, including cotton, tomato, eggplant, almonds, pistachios, pomegranate, peach, citrus, watermelon, corn, and pecan^11,19,20^.

Both *Leptoglossus* species overwinter as adults in aggregations formed most often in sheltered areas, such as evergreen trees, shrubs, and residential structures, and in the spring disperse in search of food and reproduction sites^19,21^. Sexually mature males produce a number of aldehydes, esters, and sesquiterpenes, which probably constitute an aggregation pheromone attractive to both sexes^22,23^. Olfaction is also crucial for detecting fruits or seeds through the volatiles secreted by the host plant^19^.

Antennae of insects possess a diverse set of sensory organs (sensilla) serving an array of sensory modalities such as olfaction, gustation, touch and reception of humidity and temperature^24–29^. Sensilla numbers and their morphological types/subtypes vary significantly on the insect's body. Still, all basal functional types (mechanoreception, chemoreception and thermo-/hygro-reception) are concentrated in various combinations in particular regions of the antennae^30,31^.

The function of sensilla is possible to determine based on their morphology and their distribution, but the external characteristics must be appropriately recognized and indicated. For example, sensilla with the porous wall are usually related to olfactory function, whereas mechano-, thermo- and hygro-receptors are mainly ascribed to aporous sensilla^27,28,32–35^. Moreover, the wall of porous sensilla can be a double wall or a single wall^24^. An additional characteristic to determine the sensilla's role is the type of connection between the sensillum and the cuticle surface, through the so-called inflexible socket in chemosensory sensilla and flexible socket in mechanoreceptors^26^. Olfactory sensilla are mainly localized on the antennae, and gustatory sensilla occur primarily on the labium and maxillar and mandibular palpi, rarely on the tarsi (in some insects). In contrast, the mechanosensilla and thermo-hygrosensilla have wider distribution on the insect's body^33^.

So far, the morphology and ultrastructure of the receptors in the terrestrial heteropteran taxa have been studied on several species within individual families, with particular attention paid to species harmful to agriculture or of medical importance. Most research was concerned with the typical morphological structure and function of sensilla in pentatomid bugs^29,36–43^. There are also numerous articles regarding the antennal sensilla in hematophagous species belonging to the family Reduviidae^44–49^, which showed that the type of poral system of antennal chemoreceptors has significant differences among species. Several papers concern the antennal sensory organs of the blood-sucking Cimicidae^50–52^. Antennal sensilla have also been studied in a few species of families such as Miridae^40,53–56^, Tingidae^57^, Lygaeidae^37^, Scutelleridae^58^. Within Coreoidea, sensory organs were examined in Rhopalidae^40^, Alydidae^37,59,60^, and two species of Coreidae (*Coreus marginatus* and *Leptoglossus zonatus*)^40,61,62^. Similar sets of sensilla have been recorded among the studied species, including sensilla basiconica, trichoidea, coeloconica, campaniformia and chaetica. Nevertheless, the basic types of sensilla are divided into various subtypes, and the same names often refer to entirely different sensory organs^26^ (see “Discussion”).

Initially, we began to observe the sensilla of *Leptoglossus occidentalis* with a scanning electron microscope (SEM) to identify them for a pilot study using the atomic force microscope (AFM). We chose this species due to its relatively large antennae and high material availability. The sensory organs were identified using the detailed paper of Gonzaga-Segura et al. concerning *L. zonatus*^62^. We noticed that the variety of sensilla (especially olfactory ones) in *L. occidentalis* is greater than that reported in *L. zonatus* in the study mentioned above. Moreover, the characteristics of some receptors also appeared to be slightly different. These observations led us to ask: what are the differences or similarities of sensory organs in *L. occidentalis* and *L. zonatus*?

Most heteropteran insects heavily rely on olfaction in their intra- and interspecific communication through pheromones (aggregation, sexual communication and alarm signals) and kairomones (plant volatiles), respectively^29^. The number and variety of mechano- and chemosensilla constitute an important adaptation of insects to the different habitats and colonization of new areas. Especially, the presence of different multiporous sensilla with varying organizations as regards the stimulus-transporting system at the pore level suggests an adaptation of different sensilla to specific substances (e.g., to more polar compounds or to more polar volatiles)^58,63,64^. As already mentioned, the detection of volatile substances is significant for *L. occidentalis* and *L. zonatus*. This prompted our hypothesis that a strong sense of smell contributed to their evolutionary success and their economic importance as agricultural pests and invasive species around the world.

A novel approach that combines the advantages of field emission scanning electron microscopy (FE-SEM) and atomic force microscope (AFM) was used to research micromorphological structures.

## Methods

### Materials examined

The study is based on specimens of *L. occidentalis* collected in Poland (Upper Silesia) and those of *L. zonatus* from Parlier, California (Fresno County). Due to the very low sexual dimorphism of antennal structure, it was decided to present the results without distinction by sex of specimens.

### Light microscopy

**Figure 2:**
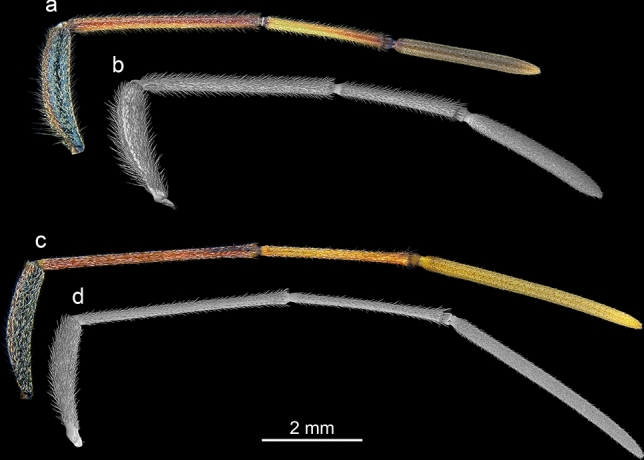
Antennae of examined species in light microscopy and SEM: *L. occidentalis* (a, b) and *L. zonatus* (c, d).

The photographs (Figs. 1, 2) were taken following the method described by Taszakowski and Kaszyca^65^. To prepare high-quality photos that would enable advanced processing (e.g., obtaining a uniform background), the specimens were glued on transparent entomological glue boards and then cleaned with a thin brush. For better visibility of details, pictures of antennae were taken with the use of a dark background. The Focus-stacked, colour photographs were captured using the following equipment: Leica M205C (stereomicroscope), Leica LED5000 HDI (high diffuse dome illumination), Leica DFC495 (digital camera), Leica application suite 4.12.0 (software) (Leica Microsystems, Vienna, Austria), Image Composite Editor (panoramic image stitcher) and Adobe Photoshop CS6 graphic editor.

### Scanning electron microscopy (SEM)

**Figure 3:**
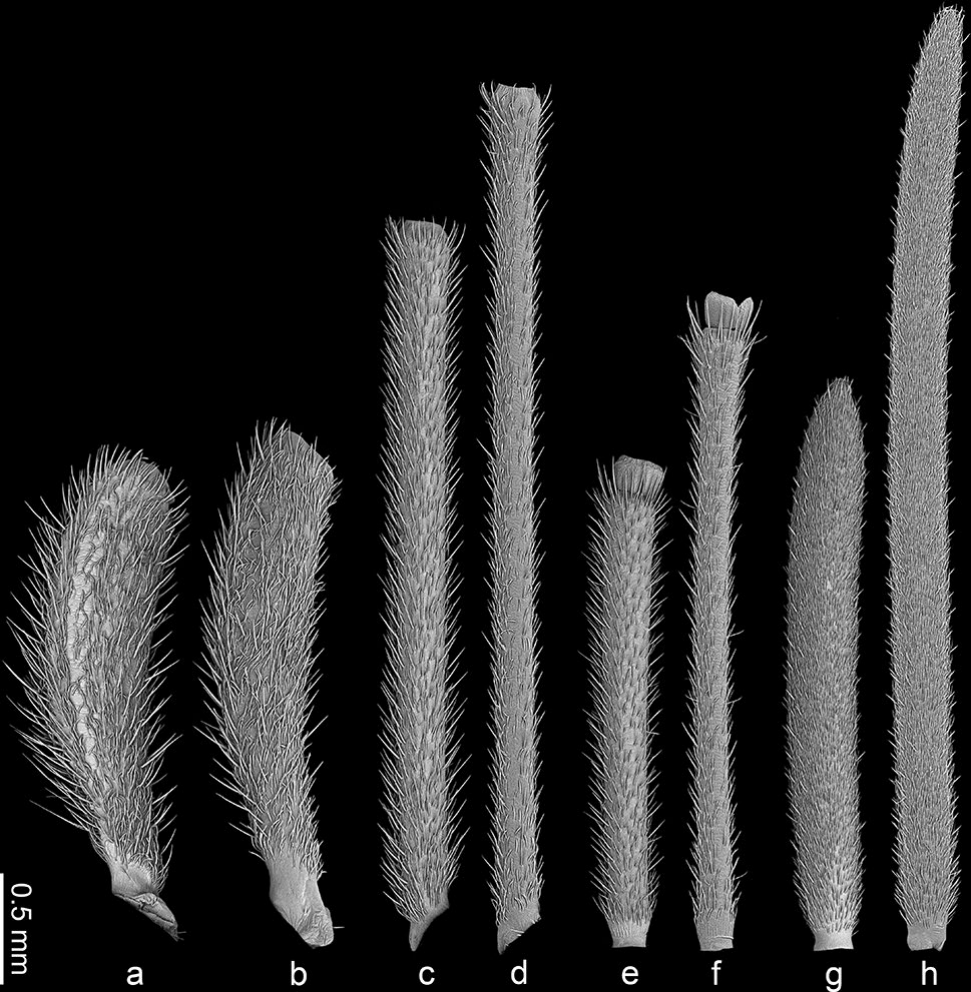
The particular antennomeres of *L. occidentalis* (a, c, e, g) and *L. zonatus* (b, d, f, h); scape (a, b), pedicel (c, d), basiflagellum (e, f), distiflagellum (g, h).

**Figure 4:**
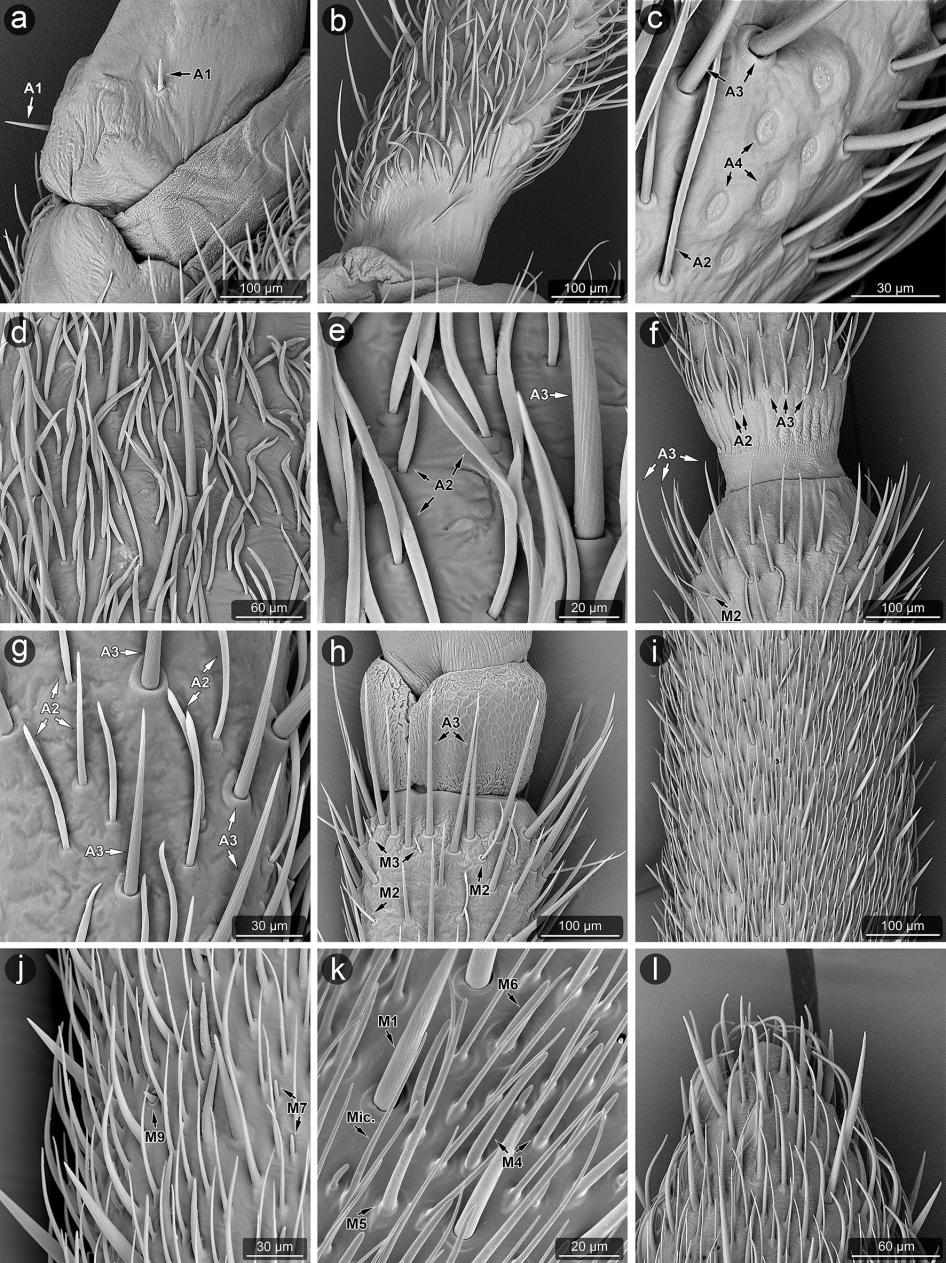
Morphology of selected parts of antennae analyzed in SEM: *L. zonatus*, the base of scape (**a**); *L. occidentalis*, the base of scape (**b**) and area with campaniform sensilla—A4 at higher magnification (**c**); *L. zonatus*, the surface of scape (**d**), as before, at higher magnification (**e**); *L. occidentalis*, connection of the scape and the pedicel (**f**); *L. zonatus*, sensilla of the pedicel (**g**); *L. zonatus*, connection of the pedicel and the basiflagellum with intercalary segment (**h**); *L. zonatus*, the surface of distiflagellum (**i**), as before, at higher magnification (**j**,**k**); *L. occidentalis*, the apex of distiflagellum (**l**).

**Figure 5:**
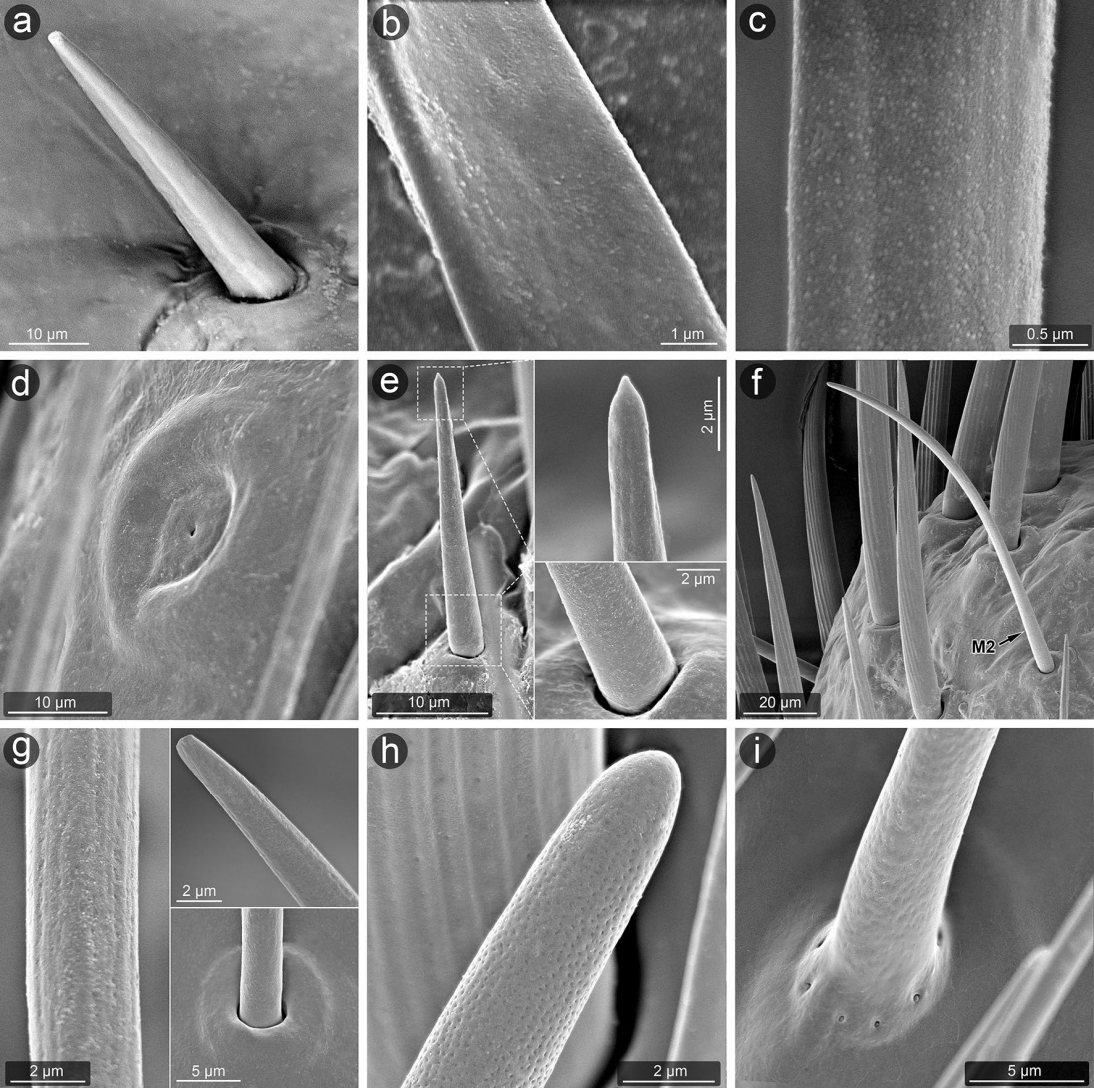
Micromorphology of selected types of sensilla (*L. occidentalis*) analysed in SEM. Type A1 (**a**); A2 (**b**); mic. (**c**); A4 (**d**); M3 (**e**); M2 (**f**,**g**); M4 (**h**); M5 (**i**). Abbreviations are given in the text.

**Figure 6:**
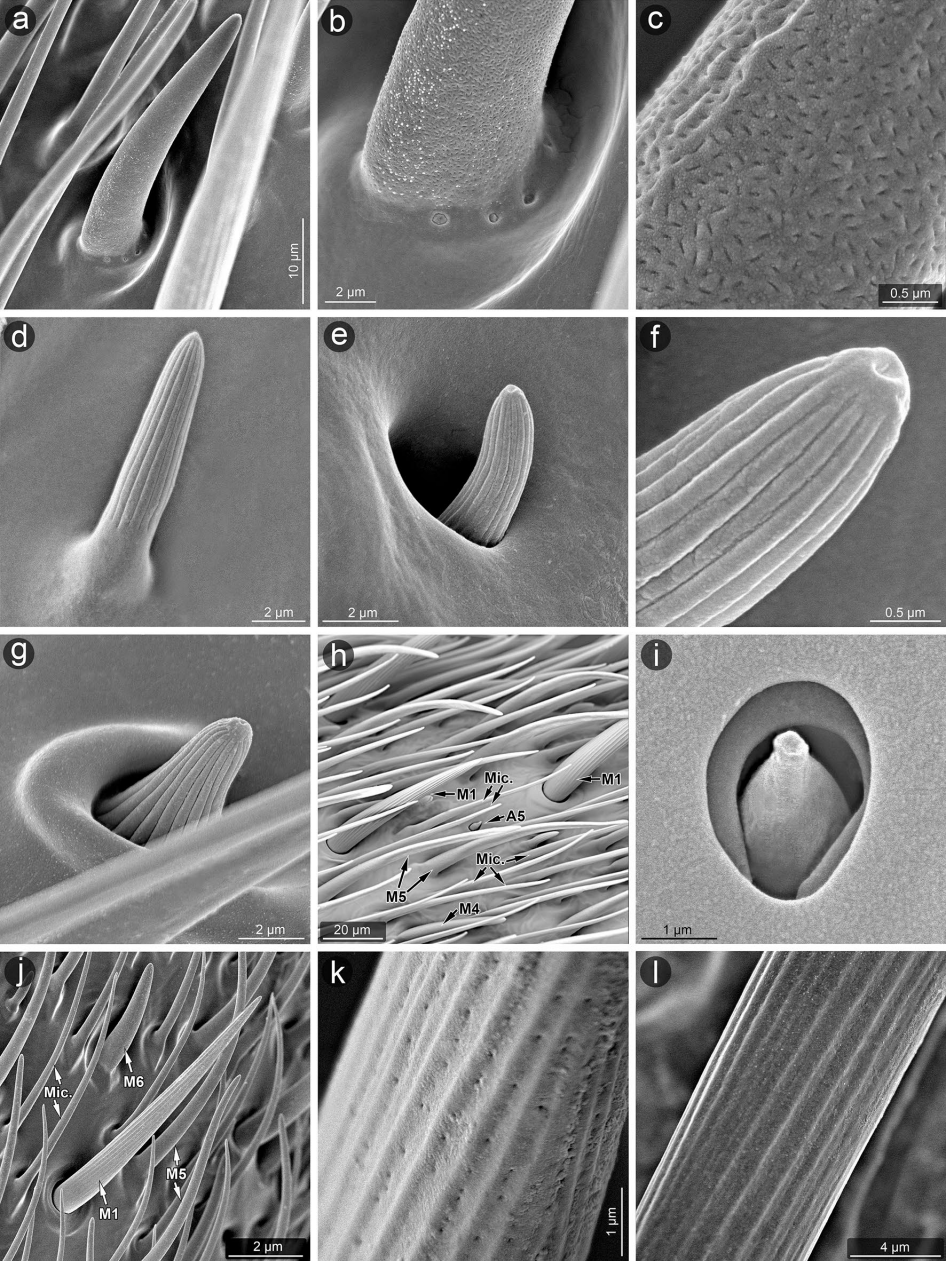
Micromorphology of selected types of sensilla (*L. occidentalis*) analyzed in SEM. Type M6 (**a**–**c**); M7 (**d**); M8 (**e**,**f**); M9 (**g**); A5 (**h**,**i**); M1 (**j**,**k**); A3 (**l**). Abbreviations are given in the text.

The material was dissected to obtain only antennae or whole heads and cleaned in detergent using an ultrasonic cleaner. Then, the standard procedure was applied^65^: dehydration with a series of baths in 80%, 90% and 96% ethanol solutions for 20 min each and two baths of 99.8% ethanol solution for 30 min each. The antennae were glued with carbon adhesive discs on the aluminium pin stubs, which then were coated with a film of gold (30 nm) using Q150T ES sputter coater with the rotary planetary stage (Quorum Technologies Ltd., Laughton, United Kingdom). SEM micrographs (Figs. 3, 4, 5, 6) were obtained using Phenom XL field emission scanning electron microscope (Phenom-World B.V., Eindhoven, The Netherlands) at 15 kV accelerating voltage and with a BackScatter Detector (BSD) and Hitachi UHR FE-SEM SU8010 (High Technologies, Tokyo, Japan) with a secondary-electron detector (ESD) at 5, 7 and 10 kV accelerating voltage. To obtain high-quality figures, fragments of antennae were imaged at high magnifications and combined using the Image Composite Editor (panoramic image stitcher) and the graphic editor Adobe Photoshop CS6. To attain the appropriate depth of field, in a few cases, a series of images at different focal distances were taken and combined using the software mentioned above.

### Atomic force microscopy (AFM)

**Figure 7:**
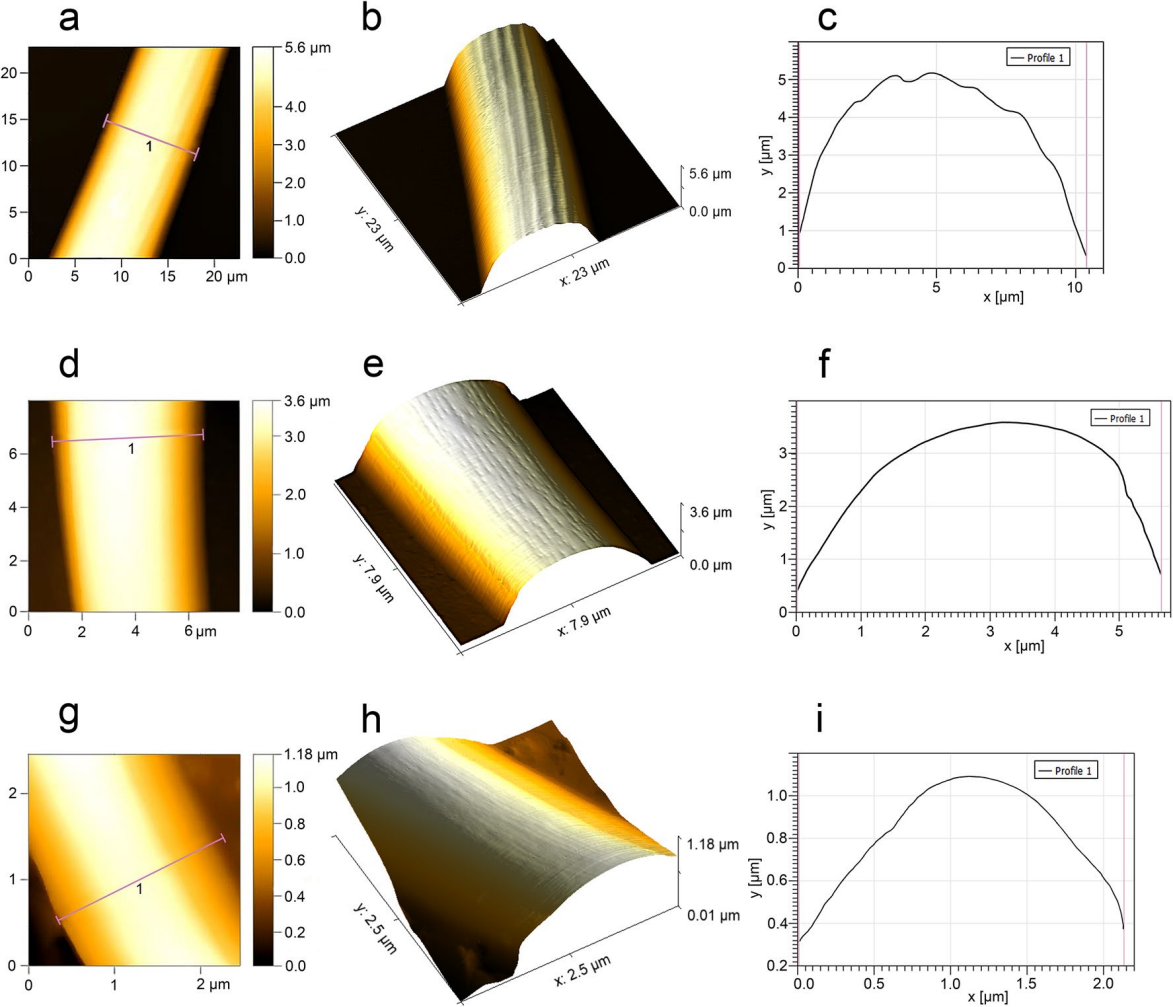
Micromorphology of selected types of sensilla (*L. occidentalis*) analyzed in AFM. Type A3: 2D view (**a**), 3D view (**b**) and surface profile (**c**); M5: 2D (**d**), 3D view (**e**) and surface profile (**f**); mic.: 2D (**g**), 3D view (**h**) and surface profile (**i**). Abbreviations are given in the text.

**Figure 8:**
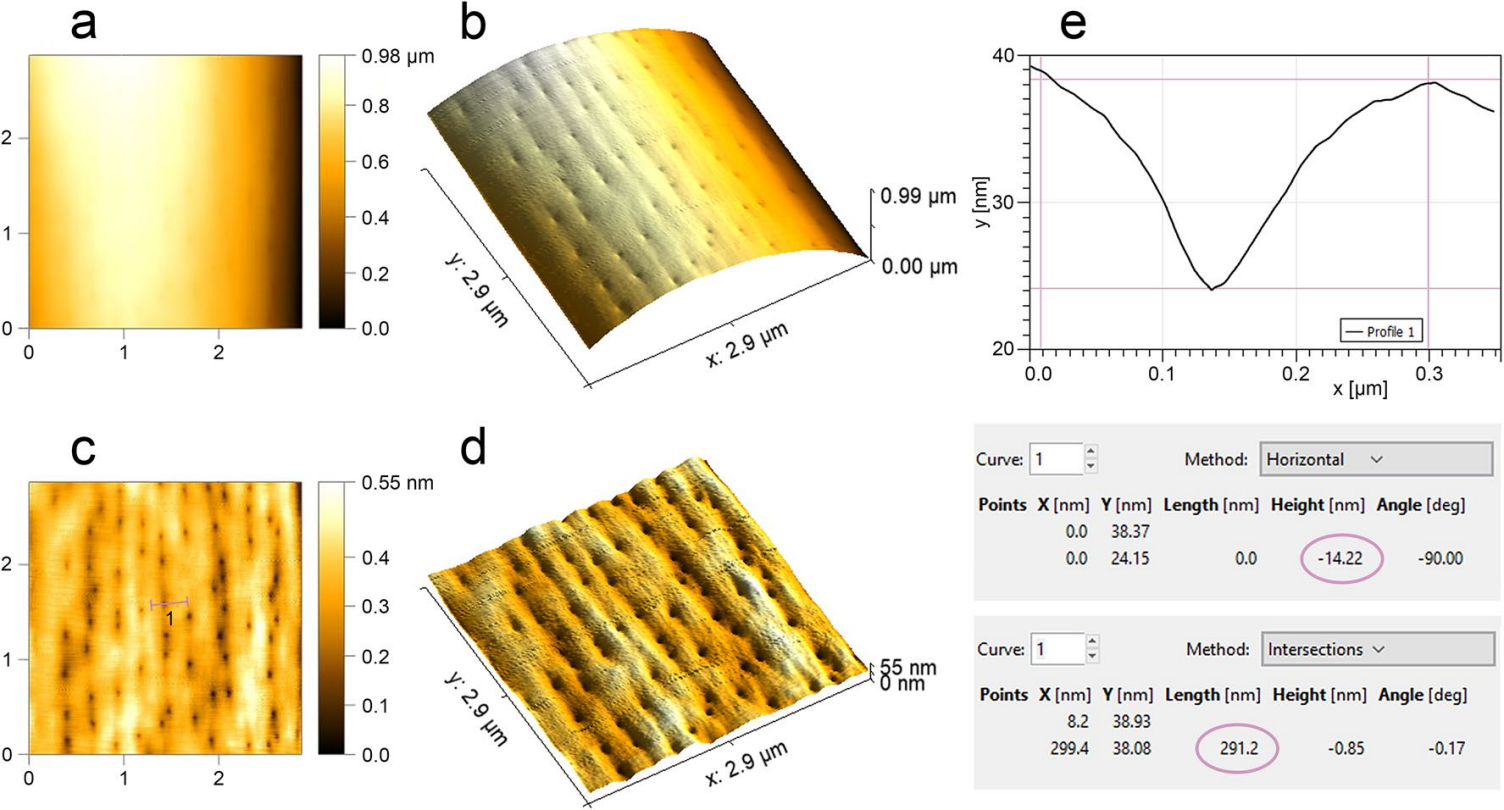
Detailed analysis of sensillum type M5 (*L. occidentalis*) in AFM: the surface of sensillum before (**a**,**b**) and after subtracting the sample curvature (**c**,**d**); profile of single pore (**e**).

A proprietary procedure for preparing research material was developed to perform analyses using an atomic force microscope (AFM). Sensilla were scraped off the antennae with a hypodermic needle (0.45 × 16 mm) and placed in a drop of ethyl alcohol (99.8%) on a microscope slide with a cavity. A perfectly smooth mica sheet surface was obtained using adhesive tape glued on top of the sheet (11 mm × 11 mm × 0.15 mm) and then torn off with a smooth movement. Then, a drop of alcohol with sensory organs was placed with a pipette on the sheet and left for 10 min (room temperature) to evaporate the alcohol. The particular sensilla on the sheet were located in SEM. Then, using their arrangement, distance from the edge and characteristic points of the preparation (e.g. some particles, scratches) by means of a stereoscopic microscope, particular sensilla was marked with a diamond scriber. Micrographs and measurements (Figs. 7, 8) of marked sensilla were prepared using CoreAFM and CoreAFM Software (Nanosurf AG Liestal, Switzerland). The Gwyddion software was used for data analysis^66^.

### Measurements

The lengths of the antennae and their particular segments were measured by Leica application suite 4.12.0 software and presented in millimetres (mm).

### Terminology for the sensilla

The identification of sensilla and the analysis of their features carried out in the present study were based on Altner and Prillinger^26^ and Shields^28^, as well as morphological comparisons of antennal sensilla of different species of Heteroptera presented in several papers^39,41–43,52,58,60,62,67^. Sensilla were divided into particular types based on the presence or absence of pores, grooved or smooth surfaces, and flexible or inflexible sockets at the base of the sensilla.

## Results

### Antennal shapes

Morphologically, the antennae (Figs. 2, 3) (the average length, n = 4 for both species) in *L. occidentalis* are shorter (12.22 mm) in comparison to *L. zonatus* (15.67 mm). In *L. occidentalis* (Fig. 3a,c,e,g), the length of the particular segments is approximately: scape (2.61 mm), pedicel (3.86 mm), basiflagellum (2.60 mm), distiflagellum (3.14 mm). In *L. zonatus* (Fig. 3b,d,f,h), the length of the segments is approximately: scape (4.29 mm), pedicel (3.79 mm), basiflagellum (3.17 mm), distiflagellum (4.71 mm).

### Sensilla types and arrangement

In the review of the sensilla types across the accessible literature, numerous classifications were based on the sensilla’s shape and size (different lengths). Primarily sensilla's terminology is used variably and interchangeably, and this is mainly the case with trichoid, chaetic and basiconic sensilla. In the current categorization of sensilla (Table 1), length is not an important criterion for classification due to significant discrepancies in the descriptions of sensilla in heteropteran species by different authors.

**Table 1:** Characteristics of the identified types of sensory organs.

Type	Figures	Pores	Cuticle	Base	Tip	Function	Remarks
A1	4a, 5a	Aporous	Smooth	Flexible socket	Blunt	Proprioception	Protruding
A2	4c–g, 5b	Aporous	Smooth	Flexible socket	Fine	Mechanoreception	Adherent
A3	4c,e–h, 6l, 7a–c	Aporous	Grooved	Flexible socket	Fine	Mechanoreception	Protruding
A4	4b,c, 5d	Aporous	–	–	–	Mechanoreception	Central moulting pore
A5	6h,i	Aporous	Smooth	Hidden in a pit	Blunt	Thermo / hygroreception	apical moulting por
M1	4k, 6h,j,k	Multiporous (wall pore)	Grooved	Flexible socket	Fine	Chemoreception	Protruding
M2	4f,h, 5f,g	Multiporous (wall pore)	Smooth	Flexible socket	Blunt	Chemoreception	Protruding
M3	4h, 5e	Multiporous (wall pore)	Smooth	Flexible socket	Sharp	Chemoreception	Protruding, apical moulting pore?
M4	4k, 5h, 6h	Multiporous (wall pore), circular pores	Smooth	Inflexible socket	Round	Chemoreception	Adherent
M5	4k, 5i, 6h,j, 7d–f, 8	Multiporous (wall pore), circular pores	Smooth	Inflexible socket	Fine	Chemoreception	Adherent, the base surrounded by gland pores
M6	4k, 6a–c,j	Multiporous (wall pore), comma-shaped pores	Smooth	Inflexible socket	Round	Chemoreception	Adherent, the base surrounded by gland pores
M7	4j, 6d	Multiporous (double wall pore)	Grooved	Inflexible socket	Round	Chemoreception	Adherent, apical moulting pore
M8	6e,f	Multiporous (double wall pore)	Grooved	Hidden in a pit	Round	Chemoreception	Apical moulting pore
M9	4j, 6g	Multiporous (double wall pore)	Grooved	Hidden in a pit	Round	Chemoreception	Apical moulting pore
Mic	4k, 5c, 6h,j, 7g–i	Aporous	Smooth	Inflexible socket	Sharp	?	Adherent

The primary description system of the antennal sensilla in *L. occidentalis* and *L. zonatus* is based on recognizing the porous and nonporous surface of the sensilla and the type of connection to the cuticle, where the flexible socket has a thin cuticular membrane, which connects the base of the sensillum with the cuticle of insect’s body and provides greater mobility at the base of the sensilla; in case of the inflexible socket, sensilla are embedded in the cuticle without a specialized membrane in the socket region.

#### Aporous sensilla with a flexible socket

##### Aporous sensillum (A1)

Smooth cone-shaped sensillum arising perpendicularly from the surface of a flexible socket. The stem of the sensillum is stiff and blunt-ended (Figs. 4a, 5a). A pair of these sensilla are present on the ventral side near the proximal end of the scape and pedicel.

##### Aporous sensillum (A2)

Smooth, flattened, hair-like process embedded in a flexible socket (Figs. 4c–g, 5b). The base of the sensillum is narrower than the distal part. These sensilla are flexible, fine ended and writhe along the antennomeres' long axes and are almost adherent to the surface. They are numerous and form irregular arrangements, mainly covering the scape, where they are most abundant. They also appear in smaller numbers on the pedicel and singly on the proximal part of the basiflagellum.

##### Aporous sensillum (A3)

The grooved wall of the hair-like process is embedded in the flexible socket (Figs. 4c,e–h, 6l, 7a–c). The half-length of the sensillum's stem is almost as wide as the base, stiff, narrowed, and fine-ended. These sensilla protrude over the surface and are directed according to the antennomeres' long axes. Sensilla A3 are singularly scattered on the surface of each antennomere except the distiflagellum. Moreover, they are numerously arranged in distal parts of the pedicel and basiflagellum.

##### Aporous sensillum (A4)

Oval and cupola-shaped structure embedded in somewhat flexible socket. These sensilla are not protruding over the surface. A group of 10 of these sensilla are located at the base of the scape. Moreover, they occur singly on the surface of antennomeres' proximal and distal ends. The sensilla are located in the parts of the antennae where deformations can be possible, predominantly near the joints (Figs. 4b,c, 5d).

##### Aporous sensillum (A5)

Is a rounded structure with a narrow and slightly flat end. The base of the sensillum is embedded in an inflexible socket. These sensilla are sunken in a shallow, oval cavity, and their shape in detail is difficult to observe. The wall pores are not visible, so this sensillum is treated as aporous (Fig. 6h,i). Very few sensilla A5 were observed on the distiflagellum of both species.

#### Porous sensilla with a flexible socket

##### Multiporous sensillum (M1)

A hair-like sensillum possesses a shallowly grooved wall. It is embedded in a flexible socket (Figs. 4k, 6h,j,k). The half-length of the sensillum process is almost as wide as the base, it is stiff and fine-ended. These sensilla are protruding over the surface, singularly scattered and directed along antennomeres' long axes. In the grooves there’re linearly arranged large oval pores. The distance between pores is significant. Sensilla M1 occur mainly on the distiflagellum.

##### Multiporous sensillum (M2)

Hair-like sensillum with almost smooth (not significantly grooved) and slightly porous wall. It is embedded in a flexible socket (Figs. 4f,h, 5f,g). The sensillum's stem is almost just as wide all along, stiff, and slightly blunted in the end. Sensilla M2 occur on basiflagellum. They’re somewhat curved and protruding over the surface (Figs. 4f,h). Numerous tiny pores are scattered on the sensillum (Fig. 5f–g).

##### Multiporous sensillum (M3)

Short cone-like sensillum with a slightly porous wall. The sensillum's stem is wider at the base, stiff, and sharp at the end. These sensilla are protruding and oriented at a high angle to the surface of the antenna (Fig. 4h). Only two sensilla M3 located on the ventral side of the distal end of basiflagellum have been observed. Tiny pores are scattered from base to end of the sensillum and are visible only in very large magnification (Fig. 5e).

#### Porous sensilla with an inflexible socket

##### Multiporous sensillum (M4)

Sensillum with smooth and distinctly porous wall embedded in the inflexible socket (Figs. 4k, 5h, 6h). Numerous rounded nano-pores are evenly distributed over the entire surface of the sensillum (Fig. 5h). The sensillum's stem is wide, stiff and rounded at the end. Sensilla M4 are almost adherent to the surface, directed along the antennomeres' long axes, and occur on the distiflagellum.

##### Multiporous sensillum (M5)

Smooth and slightly porous wall of thin, hair-like sensillum is embedded in an inflexible socket (Fig. 4k). Numerous rounded nano-pores are barely seen in the SEM (Fig. 5i), but their presence is confirmed in the AFM (Figs. 7d–f, 8). The pore diameter is 291.2 nm, and the depth is 14.22 nm (Fig. 8e). The sensillum's stem is wider at the base, stiff, and finely narrowed at the end. Around the sensillum's base, there are several gland pores. Sensilla M5 are relatively numerous on the distiflagellum, they’re curved, slightly protruding and directed along the antennomeres' long axes.

##### Multiporous sensillum (M6)

Smooth and distinctly porous wall of the cone-like sensillum is embedded in an inflexible socket (Fig. 6a,j). Numerous comma-shaped nano-pores are evenly distributed over the entire surface of the sensillum (Fig. 6a–c). The sensillum’s stem is wide, stiff and rounded at the end. Around the sensillum’s base, there are several gland pores. Sensilla M6 occur on the distiflagellum, they’re almost adherent to the surface, singularly scattered and directed according to the antennomeres' long axes.

##### Multiporous sensillum (M7)

The deeply grooved and porous wall of the cone-like sensillum is embedded in an inflexible socket (Figs. 4j, 6d). The sensillum’s stem is wide, stiff and rounded at the end. These sensilla are singularly scattered on the distiflagellum, almost adherent to the surface, and directed according to the antennomeres' long axes.

##### Multiporous sensillum (M8)

The deeply grooved and porous wall of the cone-like sensillum is probably embedded in an inflexible socket in a shallow and open cavity (Fig. 6e,f). The base of the sensillum is hidden in a cavity, but the rounded end slightly protrudes from it. These sensilla are not numerous, singularly scattered on the distiflagellum, mainly on the ventral surface.

##### Multiporous sensillum (M9)

The deeply grooved and porous wall of the cone-like sensillum is probably embedded in an inflexible socket in a shallow and open cavity (Figs. 4j, 6g). The base of the sensillum is broad and hidden in a cavity, but the rounded end slightly protrudes from it. Only a single sensillum of this type was observed on the ventral side in the middle of distiflagellum.

#### Other cuticular structures

##### Microtrichia (Mic.)

These structures are cuticular hair-like projections, slightly curved or almost straight, without morphological characteristics of a sensillum—lack of flexible sockets and wall pores (Figs. 4k, 5c, 6h,j, 7g–i). They are uniformly distributed exclusively on the distiflagellum.

## Discussion

### Shape of antennae

Representatives of the genus *Leptoglossus* possess filiform antennae located frontally on the head capsule. Both species studied are similar in size (Fig. 1)*.* However, the antennae of *L. occidentalis* are shorter (12.22 mm) in comparison to *L. zonatus* (15.67 mm). Due to their relatively large size, the antennae of these bugs are a convenient object for micromorphological research. The length proportions of particular antennomeres differ among the studied species (Figs. 2, 3). In *L. occidentalis,* the longest is pedicel; in *L. zonatus,* the longest is distiflagellum. Both species possess an intercalary segment between basiflagellum and distiflagellum. The most significant difference is observed in the length of the distiflagellum, which is significantly shorter in *L. occidentalis*. In both species, sexual dimorphism in antennal structure is weakly noticeable. According to data from Gonzaga-Segura et al.^62^, in the case of *L. zonatus*, sexual dimorphism is manifested mainly by differences in the length of the scape and pedicel. The length of both flagellomeres, on which most of the sensory organs are located, differs slightly.

### Antennal sensilla’s morphology and function

Gonzaga-Segura et al.^62^ also noted that no sexual dimorphism was found in antennal sensory organs in *L. zonatus*. To explain this fact, they proposed the hypothesis that gregarious behaviour requires the same type of sensilla in both sexes to perceive the aggregation pheromone of conspecifics. The results of our studies of antennal sensilla confirmed the lack of sexual dimorphism in *L. zonatus* and showed the same situation in the case of *L. occidentalis.* In other heteropteran insects: *Nezara viridula* (Linnaeus, 1758)^39^, *Arma chinensis* Fallou, 1881^42^, *Perillus bioculatus* (Fabricius, 1775), *Dolycoris indicus* Stål, 1876, *Plautia crossota* (Dallas, 1851)^43^ and *Halyomorpha halys* (Stål, 1855)^29^ males and females also did not exhibit any significant difference in the type, length, or the overall number of sensilla. In the case of a carefully studied species of Alydidae—*Riptortus pedestris* (Fabricius, 1775), there was no clear difference in the four main antennal sensilla types and their subtypes between males and females, except the subtype Co2, which was not found on the antennae of females.

Presented results show that the sensory organs in both species do not show significant differences. Below, we discuss the 14 types of sensilla and microtrichia (Table 1), referring primarily to the papers of Gonzaga-Segura et al.^62^ regarding sensory organs of *L. zonatus,* and Kim et al.^60^ describing the sensilla of *R. pedestris* (Table 2), and research on other, less related bugs.

**Table 2:** Comparison of the identified types of sensory organs with the results obtained for *Leptoglossus zonatus*^62^ and *Riptortus pedestris*^60^.

Type	Equivalent in *L. zonatus*	Equivalent in *R. pedestris*
A1	Small smooth trichoids	–
A2	Fattened trichoids	–
A3	Large striated trichoids (Fig. 1b) Small striated trichoids Smooth trichoids (Fig. 1b)	Chaetic sensilla, Ch1
A4	Campaniform sensilla (Fig. 2d) Placoid organs (Fig. 2e)	–
A5	–	Coeloconic sensilla, subtype Co2
M1	Small striated trichoids (Fig. 1c,f) Large striated trichoids (Fig. 1f) Smooth trichoids (Fig. 1c)	Chaetic sensilla, Ch4
M2	Basiconic sensilla, subtype 1 (Fig. 1f)	Trichoid sensilla, T3
M3	–	–
M4	Basiconic sensilla, subtype 2	–
M5	Basiconic sensilla, subtype 1 (Figs. 1c, 2a)	–
M6	Basiconic sensilla, subtype 2	Basiconic sensilla, subtype B2
M7	Basiconic sensilla, subtype 3	Basiconic sensilla, subtype B3
M8	Coeloconic sensilla	Coeloconic sensilla, subtype Co1
M9	Basiconic sensilla, subtype 4	–
Mic	Microtrichia	Trichoid sensilla, T2

Figures are also referenced for *L. zonatus* as the different names used by Gonzaga-Segura et al.^62^ refer, in fact, to the same types of sensilla.

### Mechanoreceptors

Mechanosensilla are specialised receptive organs that detect mechanical stimuli from external forces or self-movement, including air or water movements, air currents, the deformation of body regions, touch, and sound^68^.

The mechanosensilla of insects can be represented by a tremendous number of different morphological shapes^69^. Aporous sensilla with flexible sockets, presenting various lengths and shapes (bristle, trichoid, styloconic, chaetic or basiconic) consist mainly of exocuticular material and show no substructures in thin sections and cross-fractures^70^. Basic external composition of mechanosensillum includes the stem, usually with a grooved wall embedded in a flexible socket. There are also mechanosensilla with smooth surface^69^. The receptor of such sensillum consists mainly of one neuron, with unbranched dendrite terminated as a tubular body at the base of the sensillum^70^. Trichoid sensilla are the most common mechanoreceptors in insects; depending on their distribution, they can act as either exteroceptors or proprioceptors^71,72^.

Proprioception relies on mechanosensory neurons (proprioceptors) embedded in joints, muscles, and cuticle throughout the insect body^73^. Proprioceptors can be stimulated by mechanical stimuli caused by the movement of body parts^74^. These sensilla on the antennae are usually identified as short sensilla basiconica, chaetica or trichoidea, located at the junction between the antennomeres. In both species of *Leptoglossus,* there have been found sensilla (A1) that could act as proprioceptors and are identified as sensilla with a short, stiff stem and a flexible socket. Gonzaga-Segura et al.^62^ recognized a small smooth trichoid sensilla (Ssm) on *L. zonatus* between the scape and pedicel, which detects antenna's position. Similarly, in *Rhodnius prolixus* Stål, 1859 (Reduviidae), tapered hairs arising from the base of the scape and pedicel probably provide information about the relative positions of the antennomeres^44^. In other heteropteran species (stink bugs, lace bugs, water bugs, damsel bugs), these sensilla are described mainly as mechanoreceptive sensilla basiconica^31,43,57,75^. Ventura and Panizzi^59^ in *Neomegalotomus parvus* (Westwood, 1842) (Alydidae) state (rather wrong) that these sensilla are chemoreceptors. The proprioceptive sensilla are widespread in insects^33^; however, in alydid *R. pedestris,* the sensilla between the scape and pedicel are not pointed out (probably unnoticed).

In the present study, mechanosensitive role has been assigned to sensilla A2 and A3, which are aporous with a flexible socket. Their morphology differs as follows; the A2 sensillum has a smooth surface and is more adherent to the cuticular surface, whereas the A3 sensillum is grooved and protrudes above the cuticular surface. The structure of A2 sensilla is quite unusual for mechanoreceptive sensilla (flat, flexible and lying) and the exact mechanical stimuli that they could perceive are unknown. Further studies must be conducted to confirm whether this structure is in fact a sensory organ and if so, what is its exact function. As for A3 type similar aporous, striated, trichoid sensilla were identified on *Lygus lineolaris* (Palisot de Beauvois, 1818^54^). Compared to related *R. pedestris*, the A3 sensillum of *Leptoglossus* corresponds to the chaetic sensilla (Ch1)^60^. Different types of trichoid sensilla: large striated, small striated, smooth trichoids, and flattened trichoids (Fig. 1) were reported in a previous analysis of the sensilla in *L. zonatus* by Gonzaga-Segura et al.^62^. In the present study suggests that the first three of these types in *L. zonatus* are the same as mechanosensillum A3, and flattened trichoid sensilla are the counterpart to A2 (Table 2). Presumably, in the above-mentioned study, the length/size of the mechanoreceptors was analysed based on SEM micrographs, and the alignment of the sensilla at different angles influenced the size artefact and resulted in the distinction of three types of trichoid sensilla. In our study, we observed only two types of hair-like sensilla, A2 and A3, corresponding to the more numerous types/subtypes of aporous trichoid, basiconic and chaetic sensilla, described in several studied species of Heteroptera. For example; in *S. nashi* (Tingidae), the sensilla trichodea IV (long, slightly curved, with a grooved wall, narrowed and rounded tip and a flexible socket)^57^, bristle-like type 3 in *N. viridula*^39^, long straight bristle type 3 in *L. lineolaris*^54^, long sickle-shaped strong bristles (sensilla chaetica SCH) with longitudinal grooves located in an open articulating socket in *Arma chinensis*^42^ are almost identical as mechanosensilla A3.

Insects have strain sensors embedded in their exoskeleton, known as campaniform sensilla. They consist of a dome-shaped cuticular cap outside, with a dendrite of a sensory cell attached to it internally. According to Frazier^76^ and Zacharuk^77^, campaniform sensilla are mechanoreceptors. The ultrastructure of the dendritic tip region is similar to that of the trichoid sensilla^78,79^. In *Leptoglossus*, a group of dome-shaped aporous sensilla (A4) were documented that correspond to the morphological characteristics of campaniform sensilla. They are localized proximally on the ventral side of the scape. Gonzaga-Segura et al.^62^ incorrectly described this group of sensilla as placoid sensilla in *L. zonatus*. Moreover, in the studied species, singular campaniform sensilla were found scattered on other parts of antennomeres. The authors mentioned above also identified a single campaniform sensillum in both sexes of *L. zonatus*, located on the distiflagellum.

According to Kim et al.^60^, the antennae of adults of *R. pedestris* did not show presence of the campaniform sensilla. Similarly, some studies of heteropteran species, *Euschistus heros* (Fabricius, 1798*)*, *Piezodorus guildinii* (Westwood, 1837), *Edessa meditabunda* (Fabricius, 1794), *Nezara viridula* and *Probergrothius nigricornis* (Stål, 1861) (= *Odontopus nigricornis*) did not recognize the presence of campaniform sensilla on the antennae^36,41^. In contrast, these sensilla were found on the pedicel in four species of pentatomids: *Dolycoris indicus*, *Plautia crossota*, *Perillus bioculatus* and *Eocanthecona furcellata* (Wolff, 1811)^43^ and in several species from Reduviidae, Triatominae^46^. Across the insects, the campaniform sensilla usually possesses the same shape and function, although their size may differ in particular species^30^. In some other cases, a group of campaniform sensilla (9–14) were found on tibia of the cockroach, and trochanter of stick insect in which their nervous response was confirmed when loaded by self-generated or imposed forces (strains) occurring in the exoskeleton^80^.

### Thermo-hygoreceptors

In the present study, the aporous sensillum (A5) embedded in a cavity is functionally classified as thermo-hygroreceptor. Morphologically it belongs to coeloconic, aporous sensillum without a flexible socket, it is located in a chamber open to the environment according to the characteristics distinguished by Altner^63^ for this type of sensillum. Hygroreceptors occur together with thermoreceptors in pegs that have no pore system^63^. One dendrite in such an array tends to terminate underneath the peg and flatten and fold, forming interdigitated lamellae^63^. Aporous sensilla coeloconica usually are hygroreceptors, often combined with a thermoreceptive function (cold-moist-dry triad)^81^. Another characteristic is that they occur singly and rarely compared to all other types. In both *Leptoglossus* species*,* very few sensilla A5 (two or three in various specimens) were present on the distiflagellum and were omitted in the description by Gonzaga-Segura et al.^62^ in *L. zonatus*. This type morphologically corresponds to the coeloconic sensillum; subtype Co2 indicated in the *R. pedestris*^60^. Nevertheless, their live SEM observation showed that Co2 had numerous pores on the surface, which indicates chemosensory function. In some insect species, porous sensilla coeloconica have olfactory functions and may receive chemical stimuli to locate hosts or identify pheromones^27^. In some taxa of Heteroptera, *P. nigricornis* (Pyrrochoridae), *Cyclopelta siccifolia* (Westwood, 1837) (Dinidoridae), *Chrysocoris purpureus* (Westwood, 1781) (Scutelleridae), *N. viridula, P. guildinii*, *Perillus bioculatus*, *E. furcellata* and *H. halys* (Pentatomidae), thermo-hygrosensitive coeloconic sensilla were present on the flagellum^29,36,37,39,41,43^.

Compared to the abundance of hair-like sensory organs, sensilla coeloconica have been found in low numbers on the antennae of ants and other insects as well^82–88^. The peg-in-pit morphology protects the sensory peg from harsh mechanical contact, for instance, during antennal grooming, and probably avoids evaporative water loss and cooling^63,89^. Mechanical protection might be necessary for thermo-sensitive neurons whose mechanical sensitivity has previously been documented^90^. This type is common in insects and is usually present in fewer numbers or singly^91^.

### Chemoreceptors

Among the porous sensilla (olfactory), there is an enormous variation in the numbers and morphological types in different insect taxa. The reasons for this variation remain unknown, though there may be a correlation between sensillum’s morphology and the stimulus characteristics detected by the olfactory sensory neurons inside the sensillum^25^. Long sensilla trichodea sometimes are organized creating basket-like sieves to capture sex pheromone molecules, as in male moths^25^.

In the present study of *Leptoglossu*s, the porous sensilla are identified in several forms from M1–M9. However, the porous system and other morphological features differ in three (M1–M3, M4–M6 and M7–M9) distinguished sensilla groups. The first group consists of M1–M3 multiporous sensilla with flexible sockets that represent a specific morphological type. Sensilla M1 in *Leptoglossus* have grooved walls as mechanosensilla and flexible socket, but also a clearly visible pore system associated with olfactory function. Next, sensilla M2 and M3 have a non-grooved wall, relatively smooth, with rarely scattered pores similar to M1. The sensilla’s surface in M1, M2 and M3 types are not covered densely by pores as the other multiporous sensilla (M4–M6). It has been suggested by some authors that multiparous sensilla with a flexible socket might have dual mechano-olfactory function^29^ but TEM investigation of this type of sensilla didn’t show presence of tubular body at their bases (mechanosensory neurons)^58^. Therefore, sensilla M1–M3 in *Leptoglossus* probably show just olfactory function and flexible sockets allow them to move which may give them some resistance to fracturing since they seem more rigid than other multiparous sensilla (M4–M9).

Chemo- and mechanosensory function was also distinguished for contact-chemoreceptors (gustatory and tactile)^26,30^; however, in this type, there is present only one terminal pore. A similar type of sensillum’s structure (terminal pore) but with an inflexible socket and gustatory (and possibly also olfactory) function was described by Zacharuck^27^ and by Chinta et al.^54^ in *L. lineolaris*.

An apical pore was also identified on the trichoid sensilla in *N. parvus*^59^, which suggested that this sensilla may have a mechano- and chemosensory function. Gonzaga-Segura et al.^62^, did not observe the pores on striated trichoid sensilla (large and small) in *L. zonatus*. However, their shape, flexible socket, and probably unnoticed tiny pores are similar to multiporous sensillum M1. As was the case with nonporous sensilla A3, the above authors classified the same sensillum (M1) into different types (small striated, large striated and smooth trichoids), which probably resulted from not considering the perspective when assessing their size.

In contrast, Kim et al.^60^ in *R. pedestris* described the chaetic sensilla (Ch4) that can be compared with the multiporous sensillum (M1). The sensillum M2 seems similar to basiconic sensilla, subtype 1, according to Gonzaga-Segura et al.^62^, and to trichodea sensilla (T3), as stated by Kim et al.^60^. Presently distinguished type M3 is a new type concerning the data of antennal sensilla in both species of *Leptoglossus*. In the present study, the typical olfactory function can probably be indicated for the multiporous sensilla with an inflexible socket (M4 to M9).

Presently identified sensillum M4 possesses a non-grooved wall with clearly visible pores. The sensillum is basiconic, embedded in an inflexible socket, and the morphology suggests the olfactory function. The basiconic sensilla are common in heteropteran insects and are mainly categorized as olfactory. In *H. halys*, multiporous sensilla basiconica types (SB-C and SB-D)^29^ and type 2 bristle-like sensilla (well-defined socket, wall grooves with lines of minute pores, pores appear evident in cross-sections) in *Eurygaster maura* (Linnaeus, 1758)^58^, probably also represent the multiporous thin walled olfactory sensilla.

*Leptoglossus*' multiporous sensilla (M5 and M6) differ in the structure of pores which may be rounded or comma-shaped nano-pores, respectively. However, both types of sensilla possess a pore system and correspond to the thin wall multiporous sensilla described in some insects^24,26,64^. Nonetheless, in the case of sensilla M5 and M6, unspecified glands are present and open into the socket of sensillum through large pores. In the morphological study of antennal sensilla in *L. zonatus* by Gonzaga-Segura et al.^62^, such a specific characteristic was not distinguished. In the brown marmorated stink bug, *H. halys* sensillum basiconicum SB-E was described (with a smooth surface and socket of the sensillum with pores)^29^ that corresponds to the multiporous sensillum M5. Whereas in the bean bug, *R. pedestris*, basiconic sensillum subtype 2 had numerous irregular pore-like slits on the wall and a few gland pores in the socket^60^. It has an appearance similar to the multiporous sensillum M6 in *Leptoglossus*. Another case pointed out that *Telenomus busseolae* Gahan, 1922 (Hymenoptera: Platygastroidea) possesses glands that also open into the socket near the base of the gustatory sensillum^92^. Based on the comparative analysis, the group of the grooved multiporous sensilla M7, M8, and M9 can be classified as double-walled sensilla with pores^29,58^. Morphology of these sensilla (grooved, porous and embedded in an inflexible socket) show their probable olfactory function. Internally, several sensory cells within grooved double-walled pegs have branched dendrites^26^. Several papers^34,58,64^ also supported that this type of sensilla have olfactory function by investigating ultrastructure of the receptor.

The sensillum M7 of *Leptoglossus* is placed on the surface of the basi- and the distiflagellum, it’s a common sensillum present in most insect species. This sensillum was previously recognized as basiconic subtype 3^62^ and subtype B3^60^.

The sensillum M8 is similar to the M7 but partly hidden in a cavity. Like in previous data of *L. zonatus*^62^ and *R. pedestris*^60^, presently in both *Leptoglossus* species sensilla M8 are identical to the coeloconic sensilla, according to mentioned authors. Similarly, such sensillum was found in *H. halys*^29^. Type M9 is present in both studied species and in the previous study of *L. zonatus*^62^. This study's multiporous sensilla coeloconica (M8) closely resembles those observed in many other insects. Functionally, it can be taken that the grooved, double-walled sensilla contain olfactory sensory cells and additionally they may also contain cold receptive cells^26^.

Multiporous, non-grooved sensilla (M1–M6) and sensilla with grooved, double wall (M7–M9) are distributed in various patterns on the antennomeres. M1–M6 are numerous and densely cover basi- and distiflagellum, while the M7–M9 are found singularly scattered among the remaining sensilla. The different types of porous wall sensilla and their spatial distribution could be explained by different tuning of olfactory receptor neurons; that is, M1–M6 could be involved in the perception of general volatile organic compounds released by host plants, while the M7–M9 could play a role in the detection of the pheromones. The thin-walled long sensilla trichodea and thin-walled and thick, grooved wall multiporous sensilla could be an adaptation to improve olfaction, as identified in *Oncopeltus fasciatus* (Dallas, 1852) (Lygaeidae)^93^, *Oxycarenus laetus* Kirby, 1891 (Lygaeidae)^94^ and *N. parvus*^59^.

The discovery of as many as nine types of chemoreceptors indicates the studied species' highly developed sense of smell. The diversity of olfactory sensilla is probably related to the multitude of substances they use to detect^19,21–23^.

Many studies show the presence of gustatory sensilla on the antennae (usually on the terminal segments)^26,29,31,58,77^. Yet, despite thorough analysis of antennomeres' distal parts, no sensilla with terminal pore/-es were found in the studied species to which this function might be assigned.

Microtrichia are cuticle processes with smooth surface and inflexible sockets. They are morphologically similar to sensilla trichoidea^29^, to which olfactory function has been assigned but detailed studies with the use of AFM confirmed the absence of any pores therefore, they are probably different structures. On this basis, it can be concluded that microtrichia do not have a receptive function but only provide a cover for sensory organs densely located on the distiflagellum. Gonzaga-Segura et al.^62^ also suggested a lack of a sensory function of microtrichia; however, Kim et al.^60^ classify these structures of *R. pedestris* as trichoid sensilla (T2) and assign to them mechanoreceptive function.

## Conclusions

- The existing classification systems of sensory organs are often based on subjective aspects of morphology, such as their shape (trichoid, chaetic, basiconic). This makes it much more difficult to compare sensory organs, even within related taxa. The classification system should be simplified and based on clear parameters, such as the porous or nonporous surface of the sensilla and the type of connection to the cuticle.
- A comparative analysis of the sensory organs of both species did not show any differences.
- A large variety of sensory organs with olfactory functions can be recognized as one of the factors responsible for the evolutionary success of members of the genus *Leptoglossus*. These insects not only find food through their smell sense but also use it to detect pheromones and form aggregations. These abilities appear to have contributed to becoming a dangerous pest and, in the case of *L. occidentalis*, made possible to conquer numerous environments worldwide.
- Some types of sensilla (A5, M8, M9) need to be further studied anatomically in order to describe their structure and confirm their function.
- The advantages of field emission scanning electron microscopy (FE-SEM) and atomic force microscope (AFM) allow for a detailed and reliable description of micromorphological structures. However, it should be noted that using the second method is quite complicated and challenging on a larger scale.

## Data Availability

All data generated or analyzed during this study are included in this published article.
